# Comparing Robotic-Assisted to Open Radical Cystectomy in the Management of Non-Muscle-Invasive Bladder Cancer: A Propensity Score Matched-Pair Analysis

**DOI:** 10.3390/cancers15194732

**Published:** 2023-09-26

**Authors:** Etienne Courboin, Romain Mathieu, Valentina Panetta, Georges Mjaess, Romain Diamand, Gregory Verhoest, Mathieu Roumiguié, Anne Sophie Bajeot, Francesco Soria, Chiara Lonati, Claudio Simeone, Giuseppe Simone, Umberto Anceschi, Paolo Umari, Ashwin Sridhar, John Kelly, Laura S. Mertens, Rafael Sanchez-Salas, Anna Colomer, Maria Angela Cerruto, Alessandro Antonelli, Wojciech Krajewski, Thierry Quackels, Alexandre Peltier, Francesco Montorsi, Alberto Briganti, Jeremy Y. C. Teoh, Benjamin Pradere, Marco Moschini, Thierry Roumeguère, Simone Albisinni

**Affiliations:** 1 Department of Urology, University Clinics of Brussels, Hôpital Erasme, Université Libre de Bruxelles, 1070 Brussels, Belgium; gmjaess@gmail.com (G.M.); thierry.quackels@erasme.ulb.ac.be (T.Q.); thierry.roumeguere@erasme.ulb.ac.be (T.R.); albisinni.simone@gmail.com (S.A.); 2 Department of Urology, CHU Rennes, 35000 Rennes, France; romain.mathieu@chu-rennes.fr (R.M.); gregory.verhoest@chu-rennes.fr (G.V.); 3 L’altrastatistica S.R.L., Consultancy & Training, Biostatistics Office, 00100 Rome, Italy; valentina.panetta@laltrastatistica.com; 4 Department of Urology, Institut Jules Bordet, Université Libre de Bruxelles, 1070 Brussels, Belgium; romain.diamand@bordet.be (R.D.); alexandre.peltier@bordet.be (A.P.); 5 Department of Urology, Andrology and Renal Transplantation, CHU Rangueil, Paul-Sabatier University, 31000 Toulouse, France; roumiguie_mathieu@yahoo.fr (M.R.); anne-sophie_bajeot@hotmail.fr (A.S.B.); 6 Division of Urology, Department of Surgical Sciences, San Giovanni Battista Hospital, University of Studies of Torino, 10024 Turin, Italy; soria.fra@gmail.com; 7 Department of Urology, Spedali Civili di Brescia, 25123 Brescia, Italy; chiara.lonati@libero.it (C.L.); csimeone@libero.it (C.S.); 8 Department of Urology, Regina Elena National Cancer Institute, 00100 Rome, Italy; puldet@gmail.com (G.S.); umberto.anceschi@ifo.gov.it (U.A.); 9 Departement of Urology, Ospedale Maggiore della Caritá di Novara, Universitá del Piemonte Orientale, 28100 Novarra, Italy; paoloumari@gmail.com; 10 Division of Surgery and Interventional Sciences, University College London, London WC1E 6BT, UK; ashwinsridhar19@gmail.com (A.S.); john.kelly@gmail.com (J.K.); 11 Department of Urology, Netherlands Cancer Institute, 1066 CX Amsterdam, The Netherlands; ls.mertens@gmail.com; 12 Department of Urology, Institut Mutualiste Montsouris, 70123 Paris, France; raersas@gmail.com (R.S.-S.); a.colomer@gmail.com (A.C.); 13 Department of Urology, University of Verona, Azienda Ospedaliera Universitaria Integrata, 37100 Verona, Italy; mariaangela.cerruto@univr.it (M.A.C.); alxanto@hotmail.com (A.A.); 14 Department of Minimally Invasive and Robotic Urology, Wrocław Medical University, 50-556 Wroclaw, Poland; krajwoj@gmail.com; 15 Unit of Urology, Urological Research Institute, IRCCS Ospedale San Raffaele, 20132 Milan, Italy; montorsi.francesco@hsr.it (F.M.); briganti.alberto@hsr.it (A.B.); marco.moschini87@gmail.com (M.M.); 16 S.H. Ho Urology Centre, Department of Surgery, Prince of Wales Hospital, The Chinese University of Hong Kong, Hong Kong, China; jeremyteoh@surgery.cuhk.edu.hk; 17 Department of Urology, University of Vienna, 1010 Vienna, Austria; benjaminpradere@gmail.com; 18 Department of Urology, Hopital La Croix du Sud, 31000 Toulouse, France; 19 Urology Unit, Department of Surgical Sciences, Tor Vergata University Hospital, University of Rome Tor Vergata, 00100 Rome, Italy

**Keywords:** cystectomy, non-muscle-invasive bladder cancer, robotic-assisted radical cystectomy, open radical cystectomy, intracorporeal urinary diversion

## Abstract

**Simple Summary:**

In this study, we analyzed 593 patients with NMIBC who underwent radical cystectomy via a robotic-assisted or open approach between 2015 and 2020. Patients with NMIBC who underwent RARC or ORC were matched 1:1 by age, sex, BMI, year of surgery and urinary diversion. We found that RARC + ICUD for patients with NMIBC is safe and associated with a lower blood loss, a lower transfusion rate and a shorter hospital stay compared to ORC. Complication rates were similar. Concerning oncologic outcomes, RARC appeared non-inferior to ORC with no significant difference in DFS, CSS and OS. These results must be confirmed with prospective randomized studies.

**Abstract:**

Background: For non-muscle-invasive bladder cancer (NMIBC) requiring radical surgery, limited data are available comparing robotic-assisted radical cystectomy with intracorporeal urinary diversion (iRARC) to open radical cystectomy (ORC). The objective of this study was to compare the two surgical techniques. Methods: A multicentric cohort of 593 patients with NMIBC undergoing iRARC or ORC between 2015 and 2020 was prospectively gathered. Perioperative and pathologic outcomes were compared. Results: A total of 143 patients operated on via iRARC were matched to 143 ORC patients. Operative time was longer in the iRARC group (*p* = 0.034). Blood loss was higher in the ORC group (*p* < 0.001), with a consequent increased post-operative transfusion rate in the ORC group (*p* = 0.003). Length of stay was longer in the ORC group (*p* = 0.007). Post-operative complications did not differ significantly (all *p* > 0.05). DFS at 60 months was 55.9% in ORC and 75.2% in iRARC with a statistically significant difference (*p* = 0.033) found in the univariate analysis. Conclusion: We found that iRARC for patients with NMIBC is safe, associated with a lower blood loss, a lower transfusion rate and a shorter hospital stay compared to ORC. Complication rates were similar. No significant differences in survival analyses emerged across the two techniques.

## 1. Introduction

Non-muscle-invasive bladder cancer (NMIBC) accounts for 75% of bladder cancer cases [1]. Although most patients are treated with conservative therapy, radical cystectomy (RC) is recommended for NMIBC with aggressive features such as high-risk patients, very high-risk patients or patients not responsive to BCG therapy [2]. However, RC is associated with high rates of morbidity and non-negligeable mortality [3]. Robotic-assisted radical cystectomy with intracorporeal urinary diversion (iRARC) is surging worldwide in the effort to reduce morbidity [4]. Retrospective series and RCTs reported shorter lengths of hospital stays and reduced transfusions after RARC compared to open radical cystectomy (ORC) [5]. Nonetheless, no significant difference in post-operative complications have been demonstrated, whereas oncologic outcomes appear comparable [6,7,8].

While most studies comparing iRARC to ORC mainly included patients with muscle-invasive bladder cancer, no study specifically compared ORC to iRARC for NMIBC. Patients with NMIBC differ from MIBC: the tumor is theoretically strictly organ-confined, leading to a different therapeutic management even when a cystectomy is indicated. Neoadjuvant systemic therapies are not recommended for NMIBC, which exhibits a better general condition at time of surgery. The absence of an invasive tumor may lead to a more conservative treatment with organ sparing and less extended lymph node dissection [9]. Finally, NMIBC in progression to MIBC may have a worse prognosis than de novo MIBC [10].

The objective of our study is to compare the perioperative outcomes of iRARC and ORC in a multicentric cohort of patients with NMIBC using a propensity score matched-pair analysis. Furthermore, we sought to explore oncologic outcomes across the two surgical techniques for NMIBC.

## 2. Materials and Methods

### 2.1. Study Design and Data

A multicentric database of patients undergoing RC was created by the European Association of Urology-Young Academic Urologists (EAU-YAU), Urothelial Carcinoma Working Group. This multicentric cohort included patients undergoing either iRARC or ORC between 2014 and 2021. Only centers that maintained a local prospective registry of patients undergoing RC were invited to participate and they provided ethic committee and institutional review board approval. Twenty-three centers participated in the study. We then selected patients undergoing RC for high-risk and very high-risk NMIBC defined as T1 high grade (HG) associated with concomitant bladder carcinoma in situ (CIS), multiple and/or large T1HG and/or recurrent T1HG, or NMIBC that did not respond to BCG therapy defined as BCG-refractory tumor, BCG-unresponsive or BCG-relapsing [2]. This study was approved by the CNIL (Comité National Informatique et Liberté) and was conducted following the principles of the Helsinki Declaration.

### 2.2. Surgery and Perioperative Work-Up

No bowel preparation was performed prior to surgery. In the iRARC group, all urinary diversions were performed intracorporeally. All centers implemented an ERAS protocol [11]; early mobilization and oral feeding was encouraged for all patients. Low-molecular weight heparin was prescribed for four weeks following guidelines. Ureteral catheters were removed on POD 7–14 and urethral catheters (in case of neobladder) on POD 10–21. Complications were graded using the Clavien–Dindo classification (CDC) [12] and comprehensive complication index (CCI) [13]. The comprehensive complication index was calculated using an online tool (assessurgery.com (accessed on 20 January 2023)). For follow-up, patients were seen at one month post-surgery, every three months the first year, twice a year the second year, then annually.

### 2.3. Statistical Analysis

A total of 593 patients with NMIBC were available for analysis, with 409 open and 184 robotic. The propensity score was estimated using a multivariable logistic regression model. The model included the following variables: year of operation, age, sex, BMI urinary diversion in 3 classes and ASA score. After setting a caliper width of 0.25 standard deviations of the propensity score, cases were matched to controls without replacement to the closest matched propensity score with a 1:1 ratio. The standardized mean difference was used to evaluate balancing between ORC and RARC, with STD ≤ 0.1 considered to be a good balance. To take into account the matching outcome, analyses were made using robust-clustered standard errors.

Linear regression with robust-clustered standard errors (matching) was used to evaluate continuous outcomes such as operating time and blood loss. In the case of blood loss, the logarithm of the original value was used as a dependent variable and the geometric mean and SD were presented. Beta coefficients (B) and their 95% CI were reported. Logistic regression with robust clustered standard errors (matching) was used to evaluate the dichotomous outcome of either the presence of a transfusion or the presence of a complication. Odds ratios (ORs) with 95% CIs were reported. Poisson regression with robust clustered standard errors (matching) was used to evaluate quantitative outcomes such as the number of transfusions, number of complications and length of stay. Incidence rate ratios (IRRs) with 95% CIs were reported. The Mann–Whitney test was used to compare pT and pN distribution between the two groups. χ2 test was used to compare positive margins and CIS between the two groups. Kaplan–Meier curves were performed to evaluate disease-free survival (DFS), cancer-specific survival (CSS) and overall survival (OS). Cox regression robust clustered standard errors were examined to compare the two groups. Proportional assumptions were tested. A multivariable Cox regression was implemented, adding pT as independent variables. Hazard ratios (HRs) with 95% CIs were reported. A *p*-value of <0.05 was considered statistically significant. Statistical analysis was performed with STATA 16.1.

## 3. Results

A total of 143 patients operated on via iRARC were matched to 143 open cases. Table 1 shows no statistically significant difference across the two groups after propensity score matching. The stage at TURBT was comparable across the two groups (open, cT1 = 123/143 (86%), robot 126/143 (88%), *p* = 0.60), as well as the presence of CIS on TUR-BT specimens (open, CIS = 52/143 (36%), robot 58/143 (41%), *p* = 0.47). The number of patients having received previous intravesical treatment was not statistically different across the two groups (78/143, 55% patients in ORC group vs. 73/143, 51% patients in RARC group, *p* = 0.74).

Table 2 illustrates perioperative results. Operative time was significantly longer in the iRARC group (309 min vs. 288 min, *p* = 0.034). Estimated blood loss was significantly higher in the open group (471 mL vs. 279 mL, *p* < 0.001), and consequently a higher post-operative transfusion rate was observed in the open group: 29 patients (20.3%) vs. 14 patients (9.8%), *p* = 0.003. Mean length of stay was 13.7 days in the RARC group vs. 17.2 days in the ORC group, *p* = 0.007.

Concerning post-operative complications, we found no significant difference across the two groups. Similarly, early readmissions were comparable (Table 2). We found 17% for major complications in both groups; although lower in the RARC group, the comprehensive complication index did not differ significantly across the two surgical techniques. No significant difference was found in hemoglobin (*p* = 0.17) or creatinine (*p* = 0.59) change.

On final pathology (Table 3), 34.3% had muscle-invasive disease in the ORC vs. 24.5% in the RARC group (*p* = 0.069). The mean LN removed was similar between the two groups. A similar rate of lymph node-positive patients was found within the two groups (13/143 ORC vs. 15/143 RARC, *p* = 0.77). In linear regression, the number of resected lymph nodes significantly correlated to operative time (B = 1.12, 95% CI: 0.28; 1.96, *p* = 0.009). On the other hand, the number of lymph nodes did not influence blood loss (B = −0.001, 95% CI: −0.006; 0.003, *p* = 0.501) and the difference between iRARC and ORC for blood loss remained significant even when corrected for the number of lymph nodes (B = −0.23, 95% CI: −0.3; −0.1, *p* < 0.001). Estimated blood loss also increased with increasing pT stages (B = 0.04, 95% CI: 0.01; 0.06, *p* = 0.003). However, the reduced estimated blood loss observed in the iRARC group is confirmed in each pT subgroup (B = −0.22, 95% CI: −0.3; −0.1, *p* < 0.001).

**Figure 1:**
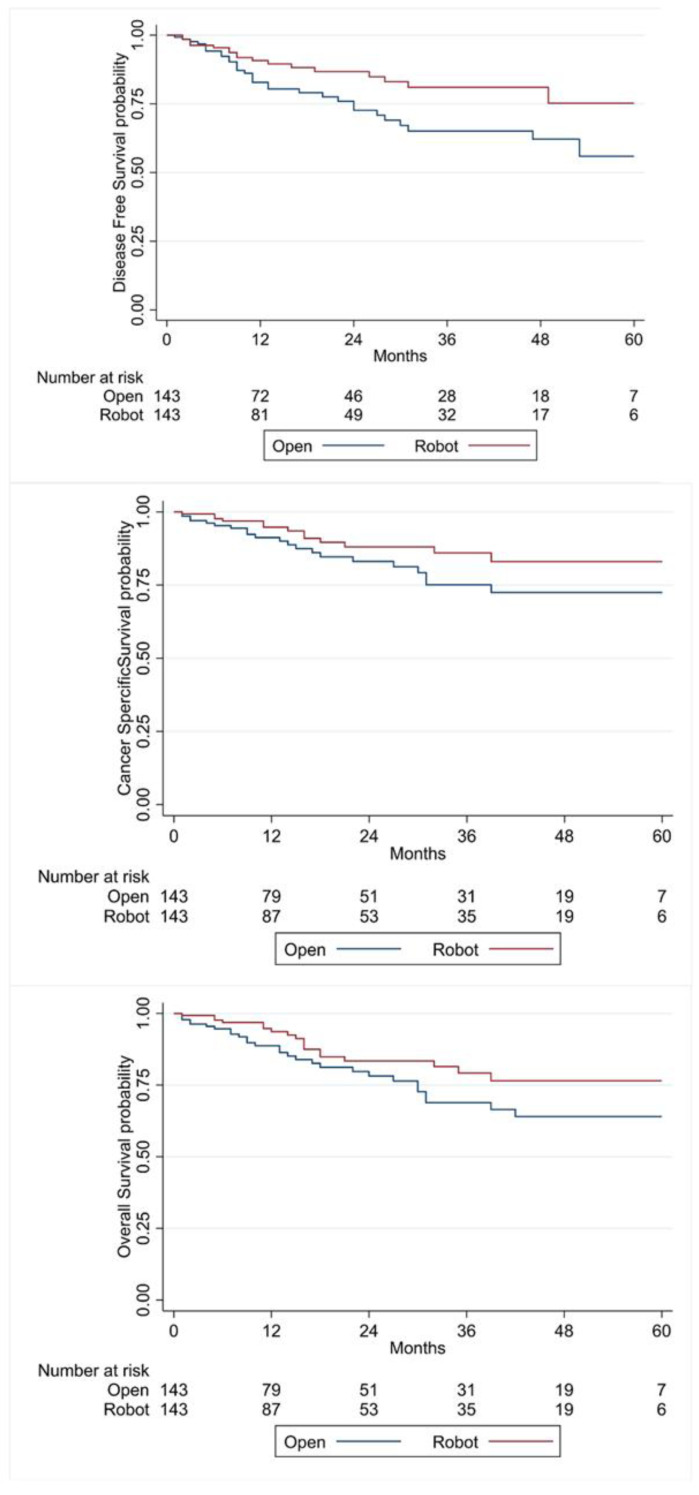
Kaplan–Meier curves for disease-free survival, cancer-specific survival and overall survival according to surgical approach.

Mean (SD) follow-up for the cohort was 20 ± 18 months for ORC and 22 ± 18 months for RARC. A total of 49 recurrences occurred, of which 31 were in ORC and 18 were in RARC. The disease-free survival at 60 months was 55.9% in ORC and 75.2% in RARC with a statistically significant difference (HR = 0.53, 95% CI: 0.29; 0.95, *p* = 0.033) in the univariate analysis (Figure 1). In Cox multivariate regression, after adjustment for the pT stage on the radical cystectomy specimen, this difference became non-significant (HR 0.56, 95% CI: 0.31; 1.02, *p* = 0.06). Forty-six patients died during follow-up, of which thirty-four related to bladder cancer. Cancer-specific (HR 0.58, 95% CI: 0.31; 1.09, *p* = 0.09) and overall survival (HR 0.59, 95% CI: 0.33; 1.04, *p* = 0.07) were not statistically different across the two surgical approaches (Figure 1).

## 4. Discussion

NMIBC is a potentially lethal disease with a major impact on patients’ lives and healthcare costs [14,15]. Choosing adequate treatment for patients can be challenging [16]. To our knowledge, this is the first comparative study on perioperative and oncologic outcomes of iRARC vs. ORC in patients with NIMBC. We reported that iRARC was associated with reduced blood loss and transfusions and reduced length of hospital stay without increased readmission rates. Nonetheless, we did not find any significant differences in post-operative morbidity, in terms of the Clavien–Dindo classification or the comprehensive complication index across the two surgical techniques. Concerning oncologic outcomes, although the DFS appeared higher in the iRARC group in univariate analysis, this is due to the increased, though not significant, >pT2 rate in the final pathology found in the ORC group.

In this study, we compared surgical outcomes across two different approaches including patients from several centers, where RC is usually carried out either by an open or a robotic approach, in an attempt to reduce selection bias. Moreover, we performed a propensity score match-pair analysis to achieve greater statistical reliability. We confirmed findings of the trials for MIBC [6,17], reporting a longer operative time (20 min) in the iRARC group, although we believe this difference is not clinically relevant. On the other hand, hospital stay was shorter (3 days) in the RARC group; although such difference appears greater than previously reported, our average LOS was longer than that of other US-based RCTs [6,7]. This must be attributed to inherent and socio-cultural differences in European and US healthcare systems.

The rate of complications was similar between the two groups, ranging from 51.8 to 59.4%. This rate is similar to those reported in MIBC-patients, for both surgical approaches as reported by Shabsigh et al. [3]. In several randomized trials [6,7], no major advantage to robotic surgery was observed regarding complication rates. Bochner et al. found no difference between the two surgical approaches (62% and 66% of RARC and ORC patients, respectively). In the RAZOR trial, there was no difference in 90 d complication, with an overall complication rate of 67% vs. 69%, respectively. In a meta-analysis by Clement et al. (including 12,640 patients comparing RARC to ORC), major complications rates were lower in the RARC group than in the ORC group [18]. On the other hand, a second meta-analysis, by Rai et al. that only included randomized trials, did not confirm these differences [19]. In the present study, we reported the comprehensive complication index for ORC and iRARC in patients with NMIBC. To our knowledge, this is the first study to explore complications for this subset of patients using the novel comprehensive index. However, no statistically significant difference was found.

The difference in DFS across the two groups deserves some reflection. Logically, the difference in pT stage (with a higher rate in >pT2 in the ORC arm, although not statistically significant) resulted in a lower recurrence-free survival rate in ORC [20]. On the other hand, the reduced transfusion rate in the RARC arm could positively affect the oncologic follow-up of patients and be associated with an improved DFS [21]. The extent of lymph node dissection can also influence oncologic outcomes: Khanna et al. published a large retrospective cohort of 1647 patients with NMIBC undergoing RC [9]. They reported a median LN count of 15 nodes, which was lower than the one reported in the present study. In multivariable analysis, a LN count >20 was associated with improved CSS (HR 0.67, 95% CI: 0.52; 0.87, *p* = 0.002) and OS (HR 0.75, 95% CI: 0.64; 0.88, *p* < 0.001). Nonetheless, in our study, the number of resected nodes (mean 21 vs. 21.3, *p* = 0.485) was not significantly different across the RARC and ORC cohorts, thus not justifying the observed difference in DFS in the univariate analysis in the present study. Another potential difference across our two matched cohorts could be the rate re-TUR, for which we are lacking data. Re-TUR has a major impact in proper patient selection before surgery [22,23]; we are unable to define whether an increased implementation of re-TUR in the iRARC arm of the present study could explain the rates of upstaging to ≥pT2 disease observed in the two arms. In a large series of cystectomies for NMIBC, Soria et al. [20] found that 50% of patients were upstaged after RC. Female gender and older age were associated with upstaging in this series; nonetheless, both factors were matched parameters in the present study and were comparable in the two groups. Iqbal et al. [24] described a large series of patients who underwent RARC for NMIBC with an upstaging rate of 31%, associated with older age, cT1 vs. cTa or Tis, and pre-operative hydronephrosis.

Our study is not devoid of limitations: First, a mean follow-up of roughly two years can be considered insufficient, especially for hard outcomes such as CSS and OS. Second, although all centers record data in a prospective manner; the retrospective design of the matched-pair analysis may increase bias. Third, quality of life and patient reported outcome measures were not available to be analyzed across the two surgical techniques. Fourth, in the present study, 47% of patients had upfront radical cystectomy, without prior intravesical therapy, and this may not reflect current practice. This elevated rate may be explained by the BCG shortage which marked the years 2015–2020 during which patients were included. Moreover, a selection bias for patients with more aggressive disease is likely: we are unable to determine the rate of variant histologies, lymphovascular invasion or other factors determining very high-risk NMIBC, which could have driven the decision for radical surgery. Finally, the absence of a standardized post-operative pathway may have contributed to the variability of post-operative morbidity. On the other hand, this is, to the best our knowledge, the largest study to date comparing iRARC to ORC for patients with NMIBC while using propensity matched-pair analysis to allow the most adequate comparison across the two surgical techniques.

## 5. Conclusions

In this propensity score matched-pair analysis, iRARC for patients with NMIBC is associated with reduced blood loss, reduced transfusions and reduced hospital stay compared to the open counterpart. However, we did not observe significant differences in surgical morbidity. The two approaches appear comparable in term of oncologic outcomes. Prospective randomized trials are needed to validate the present findings.

## Figures and Tables

**Table 1:** Baseline patient characteristics before and after matching.

	Before Propensity Score Matching					After Propensity Score Matching			
		ORC (*n* = 366)	RARC (*n* = 184)	STD	*p* Value	ORC (*n* = 143)	RARC (*n* = 143)	STD	*p* Value
Age (years)	mean (sd)	70.9 (8.4)	64.0 (10.0)	−0.73	<0.001	66.8 (8.9)	67.3 (7.6)	0.06	0.597
Male sex	*n* (%)	292 (79.8)	164 (89.1)	−0.26	0.006	122 (85.3)	128 (86.0)	−0.02	0.866
Urinary diversion					<0.001				0.909
Orthotopic neobladder	*n* (%)	75 (20.2)	94 (51.1)	0.57		62 (43.4)	59 (41.3)	−0.04	
Ileal conduit	*n* (%)	224 (61.2)	84 (45.7)	−0.31		76 (53.2)	78 (54.6)	0.03	
Ureterocutaneostomy	*n* (%)	67 (18.3)	6 (3.3)	−0.50		5 (23.5)	6 (4.2)	0.04	
ASA 3–4	*n* (%)	180 (49.2)	61 (33.2)	−0.33	<0.001	53 (37.1)	54 (37.8)	0.01	0.903
Year of surgery					<0.001				0.612
2015–2016	*n* (%)	105 (28.7)	48 (26.1)	−0.06		40 (28.0)	39 (27.3)	−0.02	
2017–2018	*n* (%)	147 (40.2)	41 (22.3)	−0.39		31 (21.7)	38 (26.6)	0.11	
2019–2020	*n* (%)	114 (31.2)	95 (51.6)	0.42		72 (50.4)	66 (46.2)	−0.08	
BMI (kg/m^2^)	mean (sd)	26.0 (4.3)	26.1 (4.2)	0.03	0.705	26.1 (5.0)	26.3 (4.4)	0.04	0.732

**Table 2:** Perioperative outcomes, complications and pathological stage.

		ORC (*n* = 143)	RARC (*n* = 143)		RARC vs. ORC	*p*
Operative time (min)	Mean (SD)	287.5 (76.6)	308.7 (83.3)	B * (95% CI)	21.12 (1.63; 40.62)	0.034
Blood loss (mL)	Mean (SD)	471.4 (2.0)	279.2 (2.6)	B * (95% CI)	−0.23 (−0.33; −0.13)	<0.001
	Median (IQR)	500 (350–650)	300 (150–585)			
Intraoperative transfusion	*n* (%)	19 (13.3)	14 (9.8)	OR ^ (95% CI)	0.71 (0.36; 1.40)	0.32
N of transfusions	Mean (SD)	1.9 (1.1)	2 (0.7)	irr ° (95% CI)	1.03 (0.76; 1.39)	0.863
	Median (IQR)	2 (1–2)	2 (2–2)			
Length of stay (days)	Mean (SD)	17.2 (12.7)	13.7 (9.0)	irr ° (95% CI)	0.80 (0.68; 0.94)	0.007
	Median (IQR)	14 (9–20)	11 (8–16)			
Post-operative transfusion	N (%)	29 (20.3)	14 (9.8)	OR ^ (95% CI)	0.32 (0.15; 0.68)	0.003
N of transfusions	Mean (SD)	2.3 (1.1)	3.2 (2.8)	irr ° (95% CI)	1.37 (0.86; 2.19)	0.188
	Median (IQR)	2 (2–3)	2 (2–3)			
Early Complications	*n* (%)	85 (59.4)	74 (51.8)	OR ^ (95% CI)	0.73 (0.5; 1.2)	0.199
Clavien—Major	*n* (%)	24 (16.8)	25 (17.5)	OR ^ (95% CI)	1.30 (0.7; 2.5)	0.448
Early Readmission	*n* (%)	14 (9.8)	16 (11.2)	OR ^ (95% CI)	1.16 (0.5; 2.5)	0.707
Comprehensive Complication Index 0–30 days	Mean SD	17.0 +/− 19.7	14.6 +/− 17.7	B * (95% CI)	−1.20 (−3.38; 0.97)	0.36
	Median (IQR)	20.6 (0–24.2)	8.7 (0–24.5)			
Late Complications	*n* (%)	28 (19.6)	23 (16.1)	OR ^ (95% CI)	0.79 (0.4; 1.5)	0.448
Clavien—Major	*n* (%)	12 (8.4)	12 (8.4)	OR ^ (95% CI)	1.5 (0.5; 4.4)	0.511
Late Readmission	*n* (%)	19 (13.3)	20 (14.0)	OR ^ (95% CI)	0.75 (0.4; 1.5)	0.398
Comprehensive Complication Index 0–90 days	Mean (SD)	20.4 (20.6)	17.4 +/− 20.8	B * (95% CI)	−1.50 (−3.91; 0.91)	0.16
	Median (IQR)	20.6 (0–29.6)	20.6 (0–29.6)			
Creatinine (mg/L)						
Pre-operative	Mean (SD)	1.1 (0.6)	1.0 (0.3)			0.081
Post-operative	Mean (SD)	1.1 (0.4)	0.9 (0.3)			0.007
Delta (Δ)	Mean (SD)	0.0 (0.4)	−0.0 (0.3)			0.59
Hemoglobin (g/dL)						
Pre-operative	Mean (SD)	13.5 (2.0)	13.4 (1.9)			0.814
Post-operative	Mean (SD)	10.6 (1.3)	10.9 (1.4)			0.130
Delta (Δ)	Mean (SD)	−2.9 (1.9)	−2.6 (1.7)			0.170
		*n*	%	*n*	%	*p*
pT stage						
	pT0	25	17.5	15	10.5	0.351
	pTa-pTis-pT1	69	48.3	93	65.0	
	pT2	14	9.8	14	9.8	
	pT3–4	35	24.5	21	14.7	
	pT2-pT4	49	34.3	35	24.5	0.069
pN stage						0.738
	pN0	125	87.4	124	86.7	
	pN1-pN3	14	9.8	15	10.5	
	pNx	4	2.8	4	2.8	
LN removed	Mean (SD)	143	21.0 (13.2)	143	21.3 (11.1)	0.485
	Median (IQR)		17 (11; 28)		19 (13; 29)	
Carcinoma in situ at RC		72	51.1	69	48.3	0.656
Positive ureteral/urethral margins		10	7.0	12	8.4	0.640

* Linear regression with robust clustered (matching) errors; ^ Logistic regression with robust clustered (matching) errors; ° Poisson regression with robust clustered (matching) errors.

**Table 3:** Pathological results.

		ORC (*n* = 143)		RARC (*n* = 143)		*p*
		*n*	%	*n*	%	
pT stage						
	pT0	25	17.5	15	10.5	0.351
	pTa-pTis-pT1	69	48.3	93	65.0	
	pT2	14	9.8	14	9.8	
	pT3-4	35	24.5	21	14.7	
	pT2-pT4	49	34.3	35	24.5	0.069
pN stage						0.738
	pN0	125	87.4	124	86.7	
	pN1-pN3	14	9.8	15	10.5	
	pNx	4	2.8	4	2.8	
LN removed	Mean (SD)	143	21.0 (13.2)	143	21.3 (11.1)	0.485
	Median (IQR)		17 (11; 28)		19 (13; 29)	
Carcinoma in situ at RC *		72	51.1	69	48.3	0.656
Positive ureteral/urethral margins		10	7.0	12	8.4	0.640

*: Radical Cystectomy.

## Data Availability

Research data are available if needed.
